# Adult stem cell and mesenchymal progenitor theories of aging

**DOI:** 10.3389/fcell.2014.00010

**Published:** 2014-03-28

**Authors:** So-ichiro Fukada, Yuran Ma, Akiyoshi Uezumi

**Affiliations:** ^1^Laboratory of Molecular and Cellular Physiology, Graduate School of Pharmaceutical Sciences, Osaka UniversityOsaka, Japan; ^2^Division for Therapies Against Intractable Diseases, Institute for Comprehensive Medical Science, Fujita Health UniversityToyoake, Japan

**Keywords:** aging, mesenchymal progenitors, adult stem cells, oxidative stress, telomere, DNA damage, niche

## Abstract

Advances in medical science and technology allow people live longer lives, which results in age-related problems. Humans cannot avoid the various aged-related alterations of aging; in other words, humans cannot remain young at molecular and cellular levels. In 1956, Harman proposed the “free radical theory of aging” to explain the molecular mechanisms of aging. Telomere length, and accumulation of DNA or mitochondrial damage are also considered to be mechanisms of aging. On the other hand, stem cells are essential for maintaining tissue homeostasis by replacing parenchymal cells; therefore, the stem cell theory of aging is also used to explain the progress of aging. Importantly, the stem cell theory of aging is likely related to other theories. In addition, recent studies have started to reveal the essential roles of tissue-resident mesenchymal progenitors/stem cells/stromal cells in maintaining tissue homeostasis, and some evidence of their fundamental roles in the progression of aging has been presented. In this review, we discuss how stem cell and other theories connect to explain the progress of aging. In addition, we consider the mesenchymal progenitor theory of aging to describing the process of aging.

## Introduction

Several theories to explain the aging-related alterations in our bodies have been proposed and accepted. The free radical theory of aging was first proposed by Harman in 1956 as one factor of aging (Harman, 1956). Aging is an universal phenomenon in living beings, and is dependent on species. Dr. Harman considered that the universality of this phenomenon meant it was caused by the same basic mechanism in all living organisms, and proposed free radicals as the causative factor. Now, the correlations between telomere length, accumulation of DNA damage, and mitochondria dysfunctions are also considered to be causative factors in aging-related alterations. Impairments of stem cell function are also supposed to explain the mechanism because stem cells are essential for maintaining tissue homeostasis, and loss of their function and/or number leads to a breakdown of function in each organ. In fact, impairments of various tissue resident stem cells during the aging process have been reported. For example, aged hematopoietic stem cells lose their engraftment potential and skew toward differentiation into myeloid cells in rodents and humans (Morrison et al., 1996; Linton and Dorshkind, 2004; Pang et al., 2011). The number of muscle stem cells (known as satellite cells) is decreased in aged mice and humans (Kadi et al., 2004; Collins et al., 2007), and some studies have indicated impaired proliferation of old satellite cells compared with young satellite cells (Bortoli et al., 2003; Conboy et al., 2005). Graying hair is a clear sign of aging in humans and many long-lived mammals (Nishimura, 2011). Melanocytes contain tyrosinase which converts tyrosine to melanin pigments in the hair during each hair cycle, but melanocyte stem cells in aged mice exhibit ectopic pigmentation or differentiation in their niche (Nishimura et al., 2005). Although accumulating evidence has demonstrated that alterations of stem cells occur during the aging process, both intrinsic and extrinsic cellular factors in the aging of adult stem cells seem to be essential in age-related phenomena (Brack et al., 2007; Pan et al., 2007).

All adult stem cells exist in a unique microenvironment, which is known as a niche. The niche is consistent with heterogeneous types of cells and extracellular matrix proteins. The blood vessel has been proposed as a niche in common. Using expression patterns of cell surface receptors of the SLAM (signaling lymphocyte activation molecule) family in hematopoietic stem cells, Kiel et al. showed that many hematopoietic stem cells are associated with sinusoidal endothelium in the spleen and bone marrow (Kiel et al., 2005). It has been reported that neural stem cells, muscle stem cells, and spermatogonic stem cells are closely associated with blood vessels (Christov et al., 2007; Yoshida et al., 2007; Tavazoie et al., 2008). In addition to blood vessels, mesenchymal progenitors (including mesenchymal stem cell and stromal cells) have recently attracted the attention of researchers in many fields. Mesenchymal progenitors are defined as cells having potential for multipotent differentiation into mesenchymal lineages including adipocytes, osteogenic, and/or chondrogenic cells. In hematopoietic stem cells, mesenchymal progenitors also serve as one of the niche cells (Omatsu et al., 2010). When mesenchymal progenitors are depleted, the number and cell size of hematopoietic stem cells are decreased. In addition, their gene expression patterns are similar to those of the phenotype of wild hematopoietic stem cells cultured without a niche. Skeletal muscle also has muscle-resident mesenchymal progenitors, which were identified as the original source of cells of pathogenic conditions such as the accumulation of adipocytes and fibrosis in muscular dystrophy (Joe et al., 2010; Uezumi et al., 2010, 2011). Although the role of mesenchymal progenitors as a niche for muscle stem cells is unknown in a steady homeostatic condition, the depletion of mesenchymal progenitors in both bone marrow and skeletal muscle leads to a loss of hematopoiesis and skeletal muscle mass in the uninjured condition (Roberts et al., 2013). Although the immunomodulation and trophic release of mesenchymal progenitors for regeneration command considerable attention (Caplan and Dennis, 2006; Le Blanc and Ringden, 2007), they also seem to play physiological roles in sustaining homeostasis of some tissues in normal conditions even without affecting the number and function of stem cells. Therefore, impairment of mesenchymal progenitors might directly contribute to the progression of aging. Mesenchymal stem cells/progenitors may be a subcategory of adult stem cells. However, in a physiological condition, at least in skeletal muscle, their contribution to generation of parenchymal cells is limited (Uezumi et al., 2010). Although contributions of bone marrow mesenchymal stem cells/progenitors to replacement of parenchymal cells have been reported, their trophic mediators seem to be the vital roles for the therapeutic effect (Caplan and Dennis, 2006). In addition, similar types of cells have been isolated from other adult tissues, including adipose, lung, skeletal muscle, salivary gland, and skin tissues (Zuk et al., 2002; Lama et al., 2007; Sudo et al., 2007; Uezumi et al., 2010). Under this criterion, mesenchymal stem/progenitors are a distinct type of stem cells from adult tissue-specific stem cells. Therefore, from this point of view, the concept of mesenchymal progenitors will be indispensable to understanding age-related alterations.

In this review, we will discuss the aged-related changes in stem cells that connect to the other theories of aging. In addition, we will discuss the possibility of a mesenchymal progenitor theory of aging based on recent research and speculations.

## Adult tissue-specific stem cells and mesenchymal progenitor cells

Adult stem cells are defined as cells having both (multiple) differentiation and self-renewal potentials. They are indispensable for renewal and regeneration of parenchymal cells after damage. In adult mammals, many but not all tissues have functional resident tissue-specific stem cells that satisfy the criteria, including hematopoietic, skeletal muscle, pigment, epithelial, sperm, adipose, intestinal, and neural stem cells. Although a subset of adult stem cells is maintained in a quiescent state (Li and Clevers, 2010), they show massive and repetitive proliferative potential in response to trauma or various stimulations. Adult stem cells exhibit global suppression of RNA polymerase II serine-2 phosphorylation, which triggers productive transcription elongation, mRNA processing, and release of mature mRNA; therefore, the transcription and translation states of adult stem cells seem to be relatively lower than those of proliferating cells (Freter et al., 2010). Even in these states, recent studies have demonstrated that a cell in a quiescent state is more likely to be in “active state” rather than in “passive state” (Cheung and Rando, 2013). In fact, canonical Notch signaling is commonly used to maintain some adult stem cells (melanocytes, neural, intestinal, and muscle stem cells), and its loss induces unusual cell cycling and/or expression of proliferative markers until eventually adult stem cell pools are exhausted (Moriyama et al., 2006; Imayoshi et al., 2010; Fukada et al., 2011, 2013; Pellegrinet et al., 2011; Bjornson et al., 2012; Mourikis et al., 2012). Although the types of Notch receptors are not yet fully understood, adult stem cells express certain Notch receptors (for example, muscle stem cells express Notch 1–3), and activated stem cells or more committed cells express the ligand (Mourikis et al., 2012; Kawaguchi et al., 2013). In this case, activated stem cells or more committed cells serve as niche cells to maintain adult stem cells. Niches are essential not only to maintain the stem cell pool, but also to regulate the cell fate of stem cells (Rompolas et al., 2013).

Like other types of niche cells, mesenchymal progenitors function in bone marrow. Mesenchymal stem cells or mesenchymal progenitors were first characterized in bone marrow, and most studies of mesenchymal progenitors are based on *in vitro* culturing. Notably, Morikawa et al. first demonstrated the prospective isolation of mesenchymal stem/progenitors in bone marrow (Morikawa et al., 2009a). In addition, our group isolated prospectively them and identified mesenchymal progenitors in murine skeletal muscle as the PDGFRα+ cell fraction (Uezumi et al., 2010, 2011, 2014). Muscle mesenchymal progenitors are also positive for CD90 and Sca-1, but negative for CD31 and CD45. Murine bone marrow-derived mesenchymal stem cells (MSCs) are also positive for PDGFRα and Sca-1 (Morikawa et al., 2009a). Mesenchymal progenitors in muscle are not derived from the somites from which myogenic cells arise because they are not labeled by *Myf5*-Cre or *Pax3*-Cre in mice (Joe et al., 2010; Liu et al., 2012). Interestingly, a subset of mesenchymal stem cells in bone marrow is labeled in *P0*-Cre mice, raising the possibility that the cells have their developmental origin in the neural crest (Takashima et al., 2007; Morikawa et al., 2009b).

**Figure 1:**
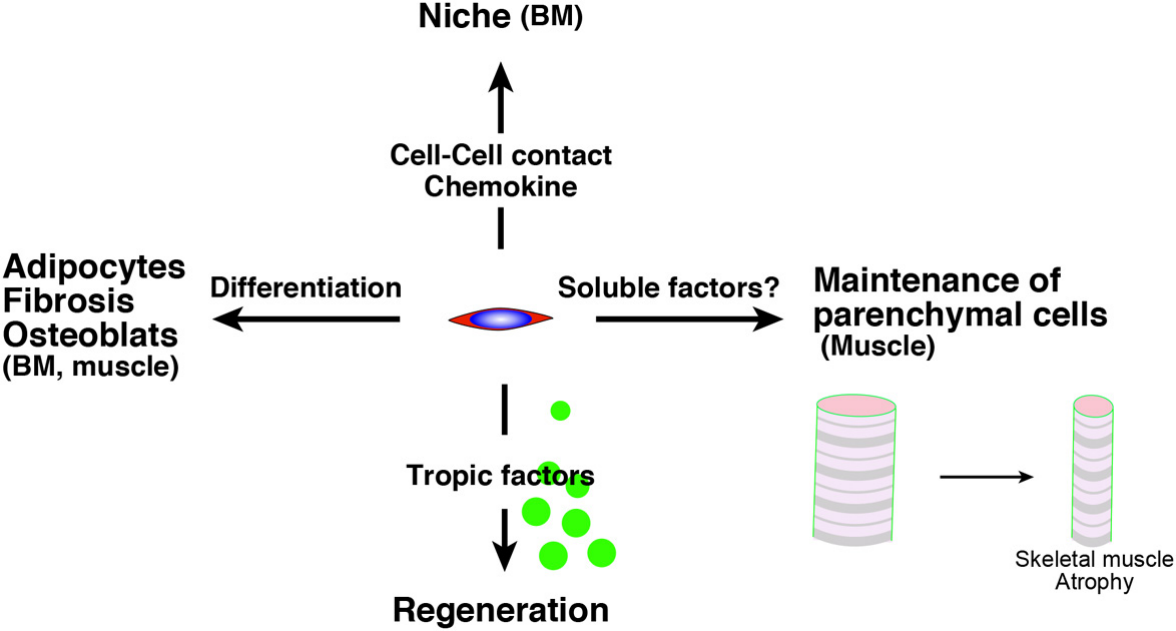
**The roles of mesenchymal progenitors.** Mesenchymal progenitors have as least four aspects. (1) The differentiative potential into adipocytes, fibrosis-related cells, osteoblast, which are observed in BM and skeletal muscle. (2) They serve as niche cells in BM through the direct cell contact or chemokine. (3) They release trophic factors to promote tissue regeneration. (4) They sustain parenchymal cells (myofiber) in skeletal muscle via unknown mechanism. BM, bone marrow.

Intriguingly, Roberts et al. demonstrated the essential roles of mesenchymal progenitors (they called them stromal cells) in the homeostasis of skeletal muscle and hematopoiesis (Roberts et al., 2013). They used fibroblast activation protein-a (FAP) as a marker of stromal cells of mesenchymal origin and inserted human diphtheria toxin receptor, firefly luciferase, and mCherry genes into mice under an FAP promoter. In these analyses, high FAP expression was observed in skeletal muscle, salivary glands, bone marrow, subcutaneous adipose, and skin. FAP+ cells co-express PDGFRα, Sca-1, and CD90. Therefore, they seem to be the ideal cell as a mesenchymal progenitor. Mice depleted of FAP+ cells by injecting diphtheria toxin exhibited a loss of skeletal muscle mass and reduction of B-lymphopoiesis and erythropoiesis. Surprisingly, two ubiquitin ligases (Atrogin-1 and MuRF1) which are essential factors for inducing skeletal muscle atrophy were upregulated in stromal cell-depleted mice 3 days after diphtheria toxin injections. This result suggests that mesenchymal progenitors have direct roles in myofibers but not in muscle stem cells. Taken together, the roles of mesenchymal progenitors are diverse, and impairment of mesenchymal progenitors is a strong candidate in the impairment of tissues, including aging (Figure 1). In the next section, we will introduce evidence for a relationship between the theory to explain aging and adult stem cells and/or mesenchymal progenitors.

## Oxidative stress and stem cells (free radical theory and stem cells)

Free radicals are unstable molecules that have an unpaired electron, and they include nitric oxide (NO^.^), some of the reactive oxygen species (ROS), and their reactive products. ROS consist of superoxide anions (O^−^_2_), hydrogen peroxide (H_2_0_2_), and hydroxyl radicals (OH^.^), which are mainly generated in cells by the mitochondrial respiratory chain (Poyton et al., 2009). Extrinsic factors (radiation, ultraviolet light, and growth factors, among others) also cause ROS production. Oxidative stress is defined as an imbalance between production of free radicals/ROS and antioxidants (Reuter et al., 2010). Among antioxidants, superoxide dismutase (SOD), catalase, peroxiredoxin, thioredoxin, and glutathione systems are known as anti-oxidative enzymes, and nuclear factor erythroid 2-related factor 2 (NRF2) is known as a master regulator of these genes (Boutten et al., 2010), which belong to the cap“n”collar (CNC) family, members of which have a conserved basic leucine zipper structure. NRF2 is regulated by KEAP1 (Kelch-like erythroid cell-derived protein with CNC homology [ECH]-associated proteins), and ubiquitination of NRF2 by KEAP1 leads to degradation of NRF2 (Itoh et al., 1999).

Hochmuth et al. demonstrated that the regulation of ROS by Keap1 and Nrf2 controls intestinal stem cell proliferation in *Drosophila* (Hochmuth et al., 2011). In young flies, CncC (a homolog of NRF2) induces antioxidant genes, which result in low oxidative stress and maintain intestinal stem cells in the quiescent state. However, Keap1 suppressed the transcriptional activities of CncC in old flies and led to deceased expression of antioxidant genes, which resulted in a high ROS and proliferative condition leading to aged-related degeneration of the intestinal epithelium. On the other hand, Tsai et al. found that murine hematopoietic stem cells and progenitor pools were expanded in *Nrf2*-knockout mice, which indicates an intrinsic dysfunction of hematopoietic stem cells in their migration and retention in the niche (Tsai et al., 2013). Although the stem cell systems and species in the two studies differ, this system might be used in some stem cell systems across species.

Forkhead box O (FOXO) is another molecule that regulates the ROS pathway in hematopoietic stem cells. The FOXO subfamily (FOXO1, 3a, 4, and 6) is known as the downstream target of the PI3K-AKT signaling pathway. Mice conditionally depleted of *Foxo1/3a/4* showed an increase in ROS and myeloid lineage expansion, lymphoid developmental abnormalities, and a decreased number of hematopoietic stem cells (Tothova et al., 2007). The authors also demonstrated that the antioxidant N-acetyl cysteine (NAC) rescues a proportion of hematopoietic stem cells in *Foxo1/3a/4*-conditional KO mice. Miyamoto et al. observed that *Foxo3a*-null hematopoietic stem cells show increased ROS levels and decreased mRNA expression of *Sod* and *catalase*. The authors also demonstrated that aged *Foxo3a*-null mice exhibited a loss of the hematopoietic stem cell pool, although young *Foxo3a*-null mice did not show such a defect (Miyamoto et al., 2007). FOXO3 is also essential in the nervous system to maintain neural stem cells (NSCs) (Renault et al., 2009). The depletion of *Foxo3* leads to decreased number of neural stem cells *in vivo*. In addition, *Foxo3*-null NSCs exhibited decreased self-renewal and an impaired differentiative potential. Using *Foxo1/3a/4*-conditional KO mice, Paik et al. also revealed the essential roles of FOXOs in maintaining the quiescent state of neural stem cells and self-renewal. *Foxo*-null neural stem cells exhibited hyperproliferation and up-regulation of ROS level, but NAC treatment does not attenuate their proliferation (Paik et al., 2009). On the other hand, the decline in self-renewal of *Foxo*-null neural stem cells was rescued by NAC treatment. The importance of ROS levels in muscle stem cells is still unknown, but Pallafacchina et al. reported that quiescent muscle stem cells express antioxidant genes at a much higher level than proliferating myoblasts do (Pallafacchina et al., 2010). They also demonstrated that quiescent muscle stem cells are more resistant to hydrogen peroxide damage than proliferating myoblasts. These results imply that adult stem cells commonly have anti-oxidative stress pathways, the dysfunction of which occurs during the aging processes in adult stem cells. Taken together, stem cell compartments seem to be directly affected in the free radical theory, in which free radicals lead to or accelerate the aging process. In addition, FOXOs and NRF2 might be major master regulators that suppress ROS in adult stem cells across species.

**Figure 2:**
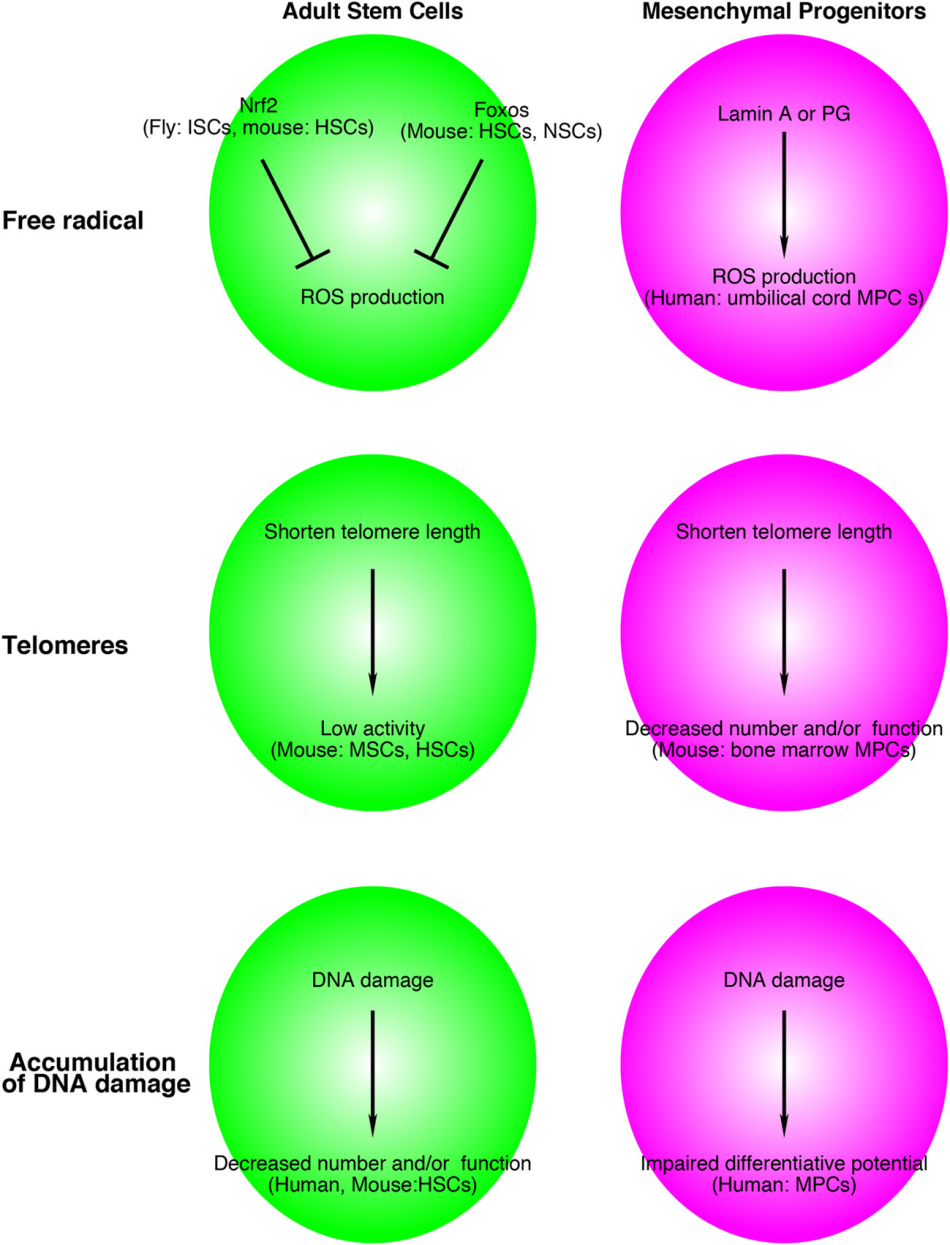
**Relationship between adult stem cell and mesenchymal progenitor theories of aging and free radicals, telomeres, and accumulation of DNA damage.** Although the supporting data are still limited, this system might be applied for aged-related alterations in adult stem cells and other tissue resident mesenchymal progenitors. The name of species, adult stem cells, mesenchymal progenitors in figures indicates experimental materials used to support these models. ISCs, intestinal stem cells; HSCs, hematopoietic stem cells; NSCs, neural stem cells; MSCs, muscle stem cells; MPCs, mesenchymal progenitors.

Intriguingly, Foxos and Nrf2 are involved in the life span of *C. elegans*. Two genes were identified as responsible for longevity in *C. elegans*. One is the *daf-2* gene, an equal homolog to both the mammalian insulin and IGF-1 receptors, and the other is *age-1*, a homolog to the worm PI3K-kinase catalytic subunit (Friedman and Johnson, 1988; Kenyon et al., 1993). In *C. elegans*, a mutation of either gene results in a profound extension of the lifespan. Insulin/IGF-1 signaling inhibits both DAF-16 (homolog of FOXO) and SKN-1 (homolog of NRF2) (Ogg et al., 1997; Tullet et al., 2008). Transgenically expressed SKN-1 prolongs the life span of *C. elegans* independently of DAF-16. Taken together, oxidative stress regulation by FOXOs and NRF2 is a conserved mechanism that contributes to the life span of stem cells and worms, and this system might be a common regulator for maintaining adult stem cells in mammalian tissues (Figure 2).

Hutchinson-Gilford progeria syndrome (HGPS) is an inherited disorder that causes premature aging and shortens the life span. The causative gene of HGPS is *PROGERIN* (*PG*), which is a truncated and farnesylated form of *LAMIN A*. HGPS affects mesenchymal linages. Zhang et al. produced iPS cells from HGPS dermal fibroblasts and differentiated them into neural progenitors, endothelial cells, fibroblasts, vascular smooth muscle, and mesenchymal stem cells (Zhang et al., 2011b). Intriguingly, *PG* was not detected in control iPS cells, but patient iPS-derived mesenchymal stem cells express high levels of PG. Scaffidi and Misteli indicated that a similar mechanism occurs in normal aging (Scaffidi and Misteli, 2006). Similar to HGPS patient cells, aged fibroblasts showed accumulation of LAMIN A/C at the nuclear lamina, decreased expression of heterochromatin protein HP1 and LAP2, and increased DNA damage compared with young cells. Mateos et al. transduced *LAMIN A* or *PG* genes in human mesenchymal progenitors derived from umbilical cord and showed that *LAMIN A* or *PG* induced high levels of ROS in human mesenchymal stem cells (Figure 2). Lentiviral induction of *LAMIN A* or *PG* genes leads to impairment of chondrogenic differentiation, which partially depends on a decrease in manganese superoxide dismutase (MnSOD) and an increase of mitochondrial MnSOD-dependent ROS (Mateos et al., 2013). Taken together, ROS accumulation in mesenchymal stem cells/progenitors might contribute to the loss of stem cells and/or tissue homeostasis in HGPS and normal aging.

## Telomeres and stem cells and mesenchymal progenitors

Characteristic repetitive DNA sequences and proteins in the end of eukaryotic chromosomes are called telomeres; telomeres are essential to maintain the stability of genomes. Telomere shortening is observed during aging of human fibroblasts (Harley et al., 1990). A DNA polymerase, named telomerase, induces telomere elongation. In order to elucidate the *in vivo* importance of telomere length, mice with the telomerase RNA component *Terc* knocked out (*Terc^−/−^*) were generated by Blasco et al. (1997). The mice show shortened telomere length, indicating that telomerase is essential to sustain telomere length. Early generations of *Terc^−/−^* mice did not show severe phenotypes, but generation 6 (G6) *Terc^−/−^* mice exhibited failures in highly proliferative organs including the hematopoietic and reproductive systems (Lee et al., 1998).

Deletion of an additional gene in *Terc^−/−^* mice produces significant changes in phenotype. For example, Wong et al. crossed *Terc^−/−^* mice with ataxia telangiectasia-mutated (Atm) knockout (*Atm^−/−^*) mice, and the offspring showed increased telomere erosion and genomic instability (Wong et al., 2003). Ataxia-telangiectasia results from mutation of the *ATM* gene and is characterized by accelerated telomere loss, genomic instability, progressive neurological degeneration, premature ageing, and increased incidence of neoplasia (Shiloh and Kastan, 2001). In addition, the frequency of T-cell lymphomas is suppressed in *Atm* and *Terc* double-knockout mice compared with *Atm^−/−^* mice, which have naturally occurring thymic lymphomas. Despite the cancer resistance of *Terc^−/−^Atm^−/−^* mice, their median life span is significantly shorter than that of *Terc^−/−^Atm^+/+^* mice.

Duchenne muscular dystrophy (DMD) is a well-known inherited X-linked disorder occurring in one in 3500 boys. The causative gene is *DYSTROPHIN*, which is essential for stability of the sarcolemma of myofibers. *Mdx* mice were discovered as mutants with high levels of muscle creatine kinase and pyruvate kinase in an inbred colony of C57BL/10 mice (Bulfield et al., 1984). Like humans, *mdx* mice have a mutation in the dystrophin gene; therefore, the *mdx* mouse is a model for human DMD. Although *mdx* mice and DMD patients have the mutation in the same gene, the clinical and pathological phenotypes of *mdx* mice are much milder than that of DMD patients. One of the differences is considered to be the great regenerative potential of muscle stem cells in *mdx* mice (Fukada et al., 2010). Sacco et al. hypothesized that the longer telomere length of *mdx* mice makes it possible for muscle stem cells to proliferate repeatedly, and generated *Terc^−/−^mdx* mice (*mdx*/mTR). Compared with *mdx* mice, *mdx*/mTR mice showed severe dystrophic phenotypes and decreased proliferative potential of muscle stem cells (Sacco et al., 2010). Unlike constitutively producing daughter cells such as hematopoietic stem cells, the proliferation of muscle stem cells during life is not very frequent. Therefore, the relationship between telomere length and aging of skeletal muscle is not clear, but these results indicate that the mechanism sustaining telomere length is one essential factor for retaining effective regeneration potential of skeletal muscles in the aged state.

As described above, many studies have shown the importance of telomere length to maintain stem cell function (Saeed and Iqtedar, 2013). In 2007, Ju et al. demonstrated that the environmental alterations due to the loss of Terc limit hematopoietic stem cell function and engraftment (Ju et al., 2007). The authors transplanted bone marrow cells from 2-month-old wild-type mice (CD45.1) into irradiated 2- and 12-month-old *Terc^−/−^* and *Terc^+/−^* littermates (CD45.2). First, they observed impaired B cell lymphopoiesis and increased myeloid proliferation in *Terc^−/−^* recipient mice. Twelve-month-old *Terc^−/−^* mice showed a more severe defect of B cell lymphogenesis and accelerated myelopoiesis compared with 2-month-old *Terc^−/−^* mice. The environment of *Terc^−/−^* mice limited the engraftment of even wild-type hematopoietic stem cells. They also showed a shortened telomere length in mesenchymal progenitors (mesenchymal stromal cells: CD45-, TER119-, and CD31- adherent cells) in *Terc^−/−^* mice and a decrease in their number. Taken together, these results suggest that the shortening of telomere length in both stem cells and mesenchymal progenitors are factors of the telomere theory aging in various tissues (Figure 2).

## Accumulation of DNA damage and tissue stem cells and mesenchymal progenitors

DNA plays essential roles in maintaining almost all functions of the body by storing and transmitting genetic information. DNA damage generally leads to changes in normal DNA structure that results in a misreading of genetic information. Among diverse types of DNA damage, oxidative DNA damage, hydrolytic DNA damage, and ultraviolet and other radiation damages are regarded as the main causes. To avoid the accumulation of DNA damage, DNA repair pathways start to work and involve numerous enzymes in this complex process. According to many studies, an obstruction mutation of the DNA repair system causes symptoms of premature aging. In humans, a mutation of *XPD* (xeroderma pigmentosum group D) is known to cause a photosensitive form of the brittle hair disorder trichothiodystrophy. A study of de Boer at al. showed that mice with mutated *Xpd* genes (*Xpd^TTD^*) exhibit many aged-related symptoms including osteoporosis, kyphosis, osteosclerosis, early graying, cachexia, infertility, and a reduced life span although the mice are born with developmentally normal phenotypes (de Boer et al., 2002). In addition, Rossi et al. showed that an *Xpd* deficiency did not result in the depletion of hematopoietic stem cells with age, but that the reconstitution and proliferative potential of hematopoietic stem cell was severely affected (Rossi et al., 2007).

The non-homologous end joining (NHEJ) pathway is a well known mechanism to repair DNA double-strand breaks. One of its components is DNA ligase IV. Using *Lig4*-deficient mice (*Lig4^Y288C^* mice), Nijnik found that impairment of the NHEJ pathway causes a progressive loss of hematopoietic stem cells during aging (Nijnik et al., 2007). Like XPD, loss of the hematopoietic stem cell pool was not observed, but functional defects of HSCs with age were reported in *Ku80* (another NHEJ component)-deficient mice (Rossi et al., 2007). In human hematopoietic stem cells and progenitors, DNA damage occurs during aging, and this accumulation of DNA is independent of telomere length (Rube et al., 2011). The relationships between DNA repair, aging, and mesenchymal progenitors remain to be elucidated. However, it has been reported at least that DNA-damaging agents such as hydrogen peroxide and paraquat lead to a loss of osteogenic differentiative potential (dexamethasone-induced ALP and mineralization) of human mesenchymal stromal cells (Alves et al., 2010) (Figure 2).

Accumulating studies also demonstrated that a component of serum is altered during aging. By utilizing parabiotic pairings, Conboy et al. showed that the age-related decline of muscle stem cell activity can be modulated by systemic factors that change with age (Conboy et al., 2005). Using young and aged (~24 months) mouse serum, same groups discovered that aged serum alters the cell fate of muscle stem cells (Brack et al., 2007). One of the candidates for the putative “aged serum” component is canonical Wnt-signaling-related molecules. Canonical Wnt signaling uses a β-catenin-dependent mechanism. Wnt signaling is an essential pathway for development and regeneration pathogenesis of many diseases, and accelerated Wnt activity is observed in different aged animal models including mice (Brack et al., 2007; Liu et al., 2007; Zhang et al., 2011a). Naito et al. identified complement C1q as a canonical activator of Wnt signaling that is upregulated in aged mice serum. They also showed that C1q can bind Frizzle (a receptor for Wnt) and activate canonical Wnt via cleavage of the ectodomain of the Wnt coreceptor LRP6 (low-density lipoprotein receptor-related protein 6) (Naito ET AL., 2012). Using rat bone marrow-derived mesenchymal stem cells, Zhang et al. demonstrated that Wnt/β-catenin signaling induced the senescence or aging in mesenchymal stem cells that accompanies DNA damage (Zhang et al., 2011a). Therefore, acceleration of the canonical Wnt signaling pathway might affect both stem cell compartments and mesenchymal progenitors in aged tissues.

## Prospects

**Figure 3:**
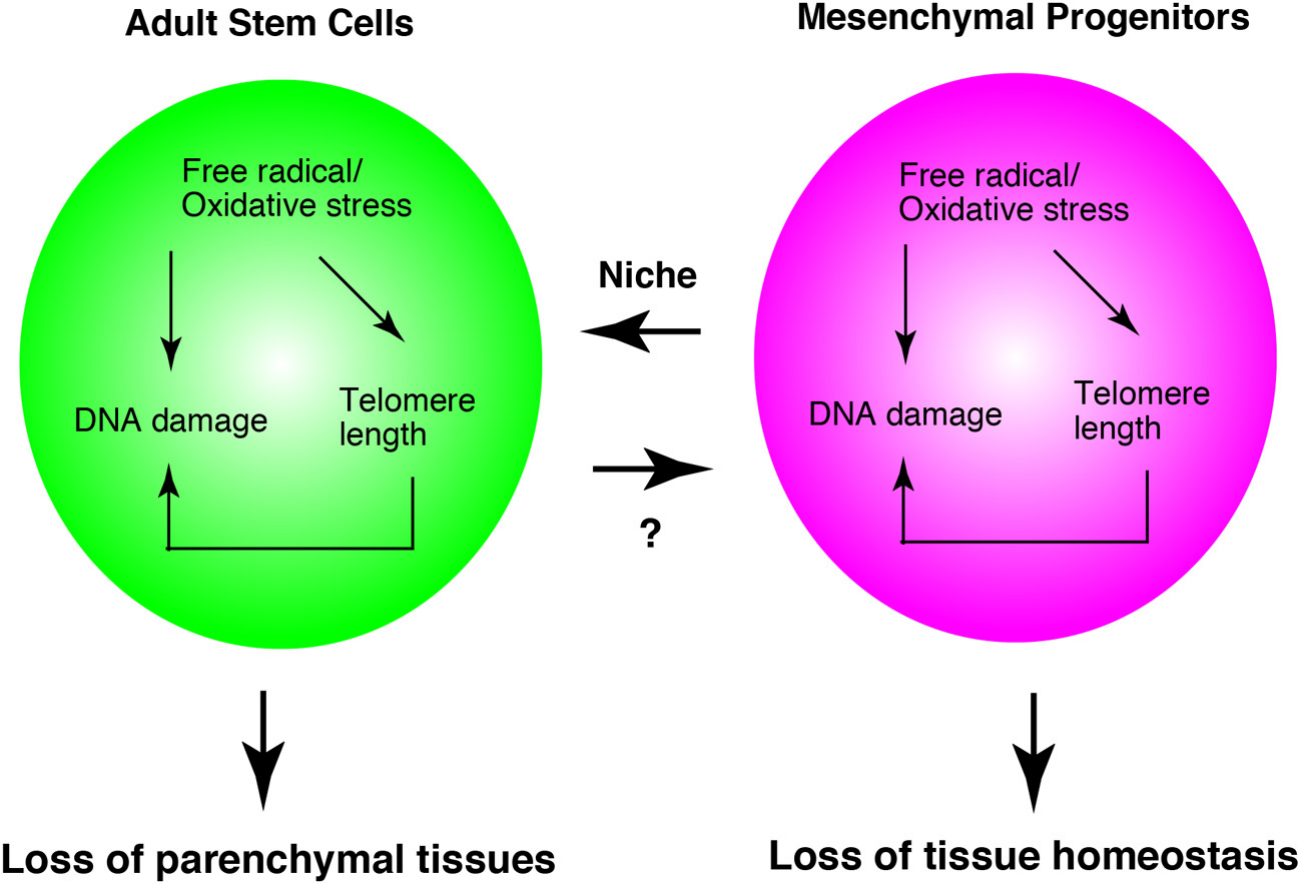
**Adult stem cell and mesenchymal progenitor theories of aging.** Interrelationship of three theories in adult stem cells and mesenchymal progenitors. Mesenchymal progenitors serve as the niche cells for hematopoietic stem cells. In skeletal muscle, mesenchymal progenitors regulate myofibers directly. Therefore, this model is based on the studies of hematopoiesis and skeletal muscle system. The appropriateness of this system in other tissues remains to be elucidated.

As described above, much evidence has shown that oxidative stress, telomere length, and DNA damage affect adult stem cells. The functions of mesenchymal progenitors are also affected by them. Oxidative stress, telomere length, and DNA damage, do not describe independent events, but they are interrelated phenomena. For example, oxidative stress leads to DNA damage (Kasai, 1997) and shortening of telomeres (von Zglinicki, 2002). Shortened telomere length in turn results in DNA damage (Vaziri and Benchimol, 1996). At least, mesenchymal progenitors serve as niche cell of hematopoietic stem cells. Therefore, the loss of mesenchymal progenitors leads to a decrease in stem cell function. In addition, dysfunction of mesenchymal progenitors might lead to a loss of tissue homeostasis without affecting adult stem cells, as was observed in skeletal muscle biology (Figure 3).

Cellular senescence is a unique state generally defined as irreversible cell cycle arrest. Recent studies have shown that senescent cells exhibit a robust increase in mRNA expression and secretion of numerous proinflammatory cytokines, which work in a paracrine manner (Campisi et al., 2011). Therefore, the chronic inflammation evoked by senescent cells is proposed as one causative factor of age-related degeneration. *p16INK4a* expression is increased in most senescent cells, and many studies showed that the age-dependent increase in *p16INK4a* expression is linked to impairment of the number and/or function of adult stem cells (hematopoietic, skeletal muscle, and nervous system) (Janzen et al., 2006; Molofsky et al., 2006; Sousa-Victor et al., 2014). Therefore cellular senescence is one mechanism of the age-related alteration of adult stem cells. In addition, *p16INK4a* expression induced senescence, and a knockdown of *p16INK4a* prevented the senescence of human mesenchymal stem cells (Shibata et al., 2007). Collectively, detrimental cellular senescence-mediated autocrine and paracrine effects contribute to “adult stem cell and mesenchymal progenitor theories of aging”; of course, it is possible that cellular senescence plays roles in differentiated cells rather than in adult stem cells and mesenchymal progenitors.

Fibroblasts can be observed in all tissues. Fibroblasts and mesenchymal progenitors show similar morphologies, but their differentiative potentials distinguish them. Sudo et al. investigated the differentiation potential of human fibroblasts derived from various tissues and found that cells originally considered fibroblasts have potential to differentiate into the mesenchymal lineage, which includes osteoblasts, chondrocytes, and adipocytes (Sudo et al., 2007). These results suggest that all tissues and organs contain mesenchymal progenitor cells. The identification of mesenchymal progenitor-derived factors and/or -expressing molecules might lead to development of anti-aging drugs that sustain the functions of aged tissues and organs. Collectively, in addition to stem cell biology, the investigation of tissue resident mesenchymal progenitors will be an essential project to sustain humans in a healthy condition for as long as possible.

### Conflict of interest statement

The authors declare that the research was conducted in the absence of any commercial or financial relationships that could be construed as a potential conflict of interest.
